# Are female adolescents in low and middle-income countries prepared for menarche?

**DOI:** 10.1016/j.amsu.2022.104658

**Published:** 2022-09-15

**Authors:** Muskan Asim Taimuri, Anusha Sumbal

**Affiliations:** Department of Internal Medicine, Dow University of Health Sciences, Mission Rd, New Labour Colony Nanakwara, Karachi City, Sindh, 74200, Pakistan

Menarche is a significant milestone in a female's life marked by the first menstrual period and the onset of the capacity to conceive [1]. Although menstruation is a natural occurrence, most girls in low and middle-income countries lack appropriate information on the physiology of menstruation and menstrual hygiene management (MHM) before menarche. This is because of the stigma and misconceptions surrounding the topic that serve as an obstacle to the healthy transfer of knowledge. Girls commonly report having no prior awareness of menstruation and only finding out about it after their first menstrual period [2].

The insufficient knowledge is coupled with various myths regarding menstruation that lead to girls associating it with negativity and fear. In a study carried out in rural Tamil Nadu, India, girls shared being told that ghosts haunt females during menstruation [3].

Females with a cloudy understanding of menstruation encounter many difficulties post menarche. These include restrained social interactions, school absenteeism and lower reproductive tract infections [[4], [5], [6]].

In most cases, mothers are the primary source of information along with sisters and other female friends [2] Unfortunately, these females themselves are ill-equipped to transfer knowledge to female adolescents. In most cases, they also do not serve as an approachable source to address ambiguities regarding menstruation or puberty. In a study carried out in Iran, it was observed that most girls are hesitant to talk to their mothers about puberty due to shame [7].

On the other hand, girls who are better prepared before menarche approach the topic with more positivity, are well aware of the physiological mechanism and can manage their menstrual hygiene more efficiently [8,9].

Thus, it becomes extremely important that young female adolescents be educated completely about menstruation and related topics. This can be achieved through a joint effort by the Government, NGOs, Health Sector, and academia.

Both the public and private sectors should concentrate on disseminating information through easily understood and accessible pamphlets or brochures that include illustrations and local language. Through this medium, information on the source and flow of menstrual blood, suitable absorbent materials, disposal methods as well as a debunking of common myths can be conveniently transmitted.

Training Programs should be organized for teachers and parents. Such programs should revolve around teaching the right ways to educate girls about menstruation as well as addressing all their concerns. Educational Institutes should also focus on eliminating the stigma affiliated with periods and for that, male students should be familiarized with the topic.

Additionally, there should be an increased emphasis on encouraging girls to seek help from health professionals if they encounter any issue related to their reproductive health rather than remaining silent. Training courses on managing the concerns raised by females who might be hesitant to consult in the first place should be provided to health professionals. Efficient management of period cramps through safe medications and heat pads should be propagated by the health and education sectors. Recent developments in MHM practices like the use of sustainable and environment friendly menstrual cups should also be introduced [10].

Such efforts have proven to be successful in the past. In an intervention study carried out in Bangladesh; after health education, significant improvement in overall menstrual practices, including improvements in using sanitary pads (22.4% change after the intervention), frequency of changing pads/cloths per day, drying the used absorbent, methods of disposal, and cleaning of genitalia were observed. The participants also reported significant improvements in their menstrual cycle regularity and fewer complications during the follow-up [11].

Such measures can hopefully pave way for a world where menstruation is regarded as a normal topic and females in all parts of the world find it easy to navigate through it.

## Ethical approval

Not applicable.

## Please state any sources of funding for your research

None.

## Author contribution

Muskan Asim Taimuri: Conceptualization, Resources, Writing- Original draft preparation, Visualization. Anusha Sumbal: Writing- Reviewing and Editing, Supervision, Project Administration.

## Please state any conflicts of interest

None.

## Consent

Not applicable.

## Registration of research studies

1. Name of the registry:

2. Unique Identifying number or registration ID:

3. Hyperlink to your specific registration (must be publicly accessible and will be checked):

## Guarantor

Muskan Asim Taimuri.

Anusha Sumbal.
